# Early pulmonary rehabilitation recommended decision-making behavior experience among pediatric intensive care unit medical staff: a qualitative study

**DOI:** 10.3389/fped.2025.1535459

**Published:** 2025-05-22

**Authors:** WenQian Cai, Meng Li, ChengCheng Li, Mei Li, XiaoKe Zhao, YaHui Zuo, Lu Zhang, YuYing Yang

**Affiliations:** ^1^ Department of Nursing, Children's Hospital of Nanjing Medical University, Nanjing, China; ^2^ Yancheng First Hospital, Affiliated Hospital of Nanjing University Medical School, Yancheng, China; ^3^ The First People's Hospital of Yancheng, Yancheng, China; ^4^ Department of Rehabilitation Medicine, Children's Hospital of Nanjing Medical University, Nanjing, China; ^5^ Pediatric Intensive Care Unit, Children's Hospital of Nanjing Medical University, Nanjing, China; ^6^ School of Nursing, Nanjing Medical University, Nanjing, China

**Keywords:** COM-B, influencing factor, pediatrics, pulmonary rehabilitation, theoretical domains framework

## Abstract

**Background:**

To understand the reasons for hindering and promoting the recommended decision-making behaviors for early pulmonary rehabilitation of PICU medical staff, and to provide a basis for developing corresponding management plans.

**Methods:**

Based on the Capability, Opportunity, Motivation-Behavior (COM-B) model and Theoretical Domains Framework (TDF), interview outlines were developed. A descriptive qualitative research method was used, and a purposive sampling method was employed to select medical staff from the intensive care unit of a tertiary children's hospital in Nanjing from September to December 2023 for semi-structured interviews. The interview data were coded using the COM-B and TDF frameworks, and analyzed, summarized, and refined using the Colaizzi 7-step method to extract themes.

**Results:**

Four main themes and 13 sub-themes were extracted, including the need for pulmonary rehabilitation knowledge and skills, the experience of implementing pulmonary rehabilitation in critically ill children, communication and collaboration in the PICU rehabilitation platform, and external support for PICU pulmonary rehabilitation.

**Conclusion:**

In the process of recommending early pulmonary rehabilitation for critically ill children, departments should help PICU medical staff change their views on pulmonary rehabilitation and acquire relevant knowledge and skills, strengthen multidisciplinary cooperation, optimize external support, and create a good practice environment for the implementation and promotion of early pulmonary rehabilitation for critically ill children.

## 1 Introduction

The advancements in pediatric critical care management have significantly improved survival rates for children in the Pediatric Intensive Care Unit (PICU) and reduced mortality rates. However, factors such as mechanical ventilation, the use of sedatives and analgesics, and immobilization can lead to more than half of critically ill children experiencing Pediatric Post-Intensive Care Syndrome (p-PICS). This condition not only affects the course of their illness but also impacts long-term functional outcomes, resulting in a decline in the quality of life for these children (1, 2). As a result, prompt and efficient intervention strategies are essential for children in critical condition. Pulmonary rehabilitation (PR) is an all-encompassing method that includes exercise training, education, and changes in behavior. The aim is to improve the physical and mental health of patients while encouraging lasting commitment to health-promoting habits (3). Research on critically ill adult patients indicates that early pulmonary rehabilitation is linked to improved outcomes (4). However, the prevalence of pulmonary rehabilitation among critically ill children is low, and there is limited research focusing on pulmonary rehabilitation practices for this population. This may be attributed to several obstacles that hinder the integration of pulmonary rehabilitation into the daily activities of the PICU (5). Previous quantitative research has identified several factors that influence the implementation of early rehabilitation in PICU. These factors include the heterogeneity of children's ages and cognitive abilities, the absence of rehabilitation guidelines specifically for critically ill children, limited availability of rehabilitation resources, and caregivers' lack of awareness regarding the benefits and significance of early rehabilitation for this vulnerable population (6). Among these professionals, healthcare workers are the frontline personnel responsible for the care of children and the implementation of early PR for critically ill patients. They play a crucial role in decision-making and recommendations regarding PR treatments, and their understanding of these practices forms the foundation for effective rehabilitation in critically ill children. Therefore, this study aims to explore the barriers and facilitators influencing the decision-making behavior related to early PR recommendations among healthcare professionals in PICU. By adopting a qualitative perspective, the study seeks to gain a deeper understanding of the diseases, experiences, and behaviors involved, ultimately providing a reference for developing early PR programs for critically ill children.

## 2 Methods

### 2.1 Design

We conducted a qualitative, exploratory study using a semi-structured, one-on-one interview method. This study follows the Standards for Reporting Qualitative Research (SRQR) (Supplementary Appendix 1) to ensure transparency in the research report (7). This study was approved by the Research Ethics Committee of the Children's Hospital of Nanjing Medical University (approval number 202405014-1).

### 2.2 Conceptual model

To provide theoretical and practical guidance for this study, we utilized two complementary frameworks: the Capacity, Opportunity, Motivation-Behavior (COM-B) model and the Theoretical Domains Framework (TDF). The COM-B model, along with intervention functions and policy categories, is situated within the behavior change framework, aiming to identify deficiencies in target behaviors and summarize the factors that influence behavior (8). TDF is an integrated theoretical framework. In 2005, Michie et al. (9) systematically reviewed 33 behavior change theories and consolidated them into 12 domains within the TDF. In 2012, Cane et al. (10) revised the framework to include 14 theoretical domains: knowledge, skills, social/professional role identity, beliefs about capabilities, optimism, beliefs about consequences, reinforcement, intentions, goals, memory/attention and decision processes, environment context and resources, social influences, emotions, and behavior regulation. These domains encompass individual, organizational, and societal levels, with the goal of establishing a comprehensive theory of behavior change to inform behavioral intervention research. The TDF can support the COM-B model in identifying specific and comprehensive factors related to target behaviors, with each domain of the TDF corresponding to a specific component of the COM-B model (11). These two theoretical frameworks have been effectively utilized to identify barriers and facilitators related to behavior, and in many instances, to design interventions (12, 13).

### 2.3 Setting, participants and sampling

This study was conducted at a tertiary children's hospital located in Nanjing, Jiangsu Province, China. Using purposive sampling, medical staff from PICU were selected as interview subjects. Inclusion Criteria: Participants must have a minimum of three years of work experience in PICU, as well as experience in rehabilitation training and practical rehabilitation for critically ill children. Informed consent and voluntary participation in this study are also required. Exclusion Criteria: Individuals who are rotating or in training positions, as well as those who are not on duty during the survey period due to sick leave, travel abroad, or other reasons, will be excluded from the study. The sample size will continue to be collected until data saturation is achieved, indicating that no new themes are emerging.

### 2.4 Date collection

Our research team comprises seven members: one chief nurse with 30 years of clinical nursing and nursing management experience, one deputy chief nurse with 15 years of rehabilitation nursing experience, one nurse supervisor with expertise in qualitative research, and four nursing graduate students who have completed training in qualitative research courses. Team members created a preliminary interview outline utilizing the COM-B and TDF frameworks by reviewing evidence from both domestic and international literature and consulting with clinical experts. We selected three medical staff members for preliminary interviews. Based on the results of these interviews, we optimized and finalized the interview outline. The complete interview guide can be found in Supplementary Appendix 2.

The interview took place in the PICU office. Prior to the interview, the interviewer introduced themselves, explained the purpose of the research, informed the interviewee that the entire process would be recorded, and provided participant information and informed consent forms. Consent was obtained and signed upon agreement. Each interview was conducted by the first author, with durations ranging from 20 to 50 min. The interview was considered complete when all key points outlined in the interview guide had been discussed and the interviewee had no further questions. Within 24 h after the interview, two researchers independently used iFlytek voice transcription software to transcribe the recorded audio verbatim into text.

### 2.5 Date analysis

We used NVivo.11 software to organize and analyze the transcribed text. First, two researchers independently and repeatedly read the transcribed text to familiarize themselves with the data. They employed Colaizzi's seven-step phenomenological analysis method for initial coding to generate initial themes (14). Then, identify sub-themes related to the initial theme, and subsequently map the initial codes back to the TDF according to the categories of barriers and facilitators. In certain instances, these codes may be mapped to multiple domains and summarized using the more straightforward COM-B model. In addition, the frequency percentage of each TDF theoretical domain categorized as barriers and facilitators under each theme was calculated separately to assess their significance. The coding results were cross-checked, and any discrepancies were discussed and resolved with a third researcher.

## 3 Results

### 3.1 Demographic characteristics

A total of 14 healthcare professionals from PICU participated in the interview study. This group included 6 registered nurses (comprising 2 nursing managers, 2 critical care specialty nurses, and 2 registered nurses), 5 PICU physicians, and 3 rehabilitation therapists. The coding of participants was organized based on the order of the interviews, with “N” representing nurses, “D” for doctors, and “T” for therapists. Detailed demographic information about the respondents can be found in Table 1.

**Table 1:** General information of respondents (*n* = 14).

Number	Gender	Age	Title	Education level	Years in PICU	Interview time
N1	F	39	Supervisor nurse	Undergraduate	18	33
N2	M	36	Supervisor nurse	Undergraduate	14	43
N3	F	32	Senior nurse	Undergraduate	12	30
N4	F	30	Senior nurse	Undergraduate	7	23
N5	F	41	Supervisor nurse	Undergraduate	16	40
N6	F	38	Supervisor nurse	Master	11	36
D1	F	29	Resident physician	Master	4	31
D2	M	33	Attending physician	Master	9	44
D3	F	38	Attending physician	Doctor	10	31
D4	M	35	Attending physician	Master	9	29
D5	M	42	Deputy chief physician	Doctor	12	23
T1	M	40	Supervisor technologist	Undergraduate	13	28
T2	M	32	Supervisor technologist	Master	6	36
T3	F	38	Supervisor technologist	Master	7	44

### 3.2 Themes

**Figure 1:**
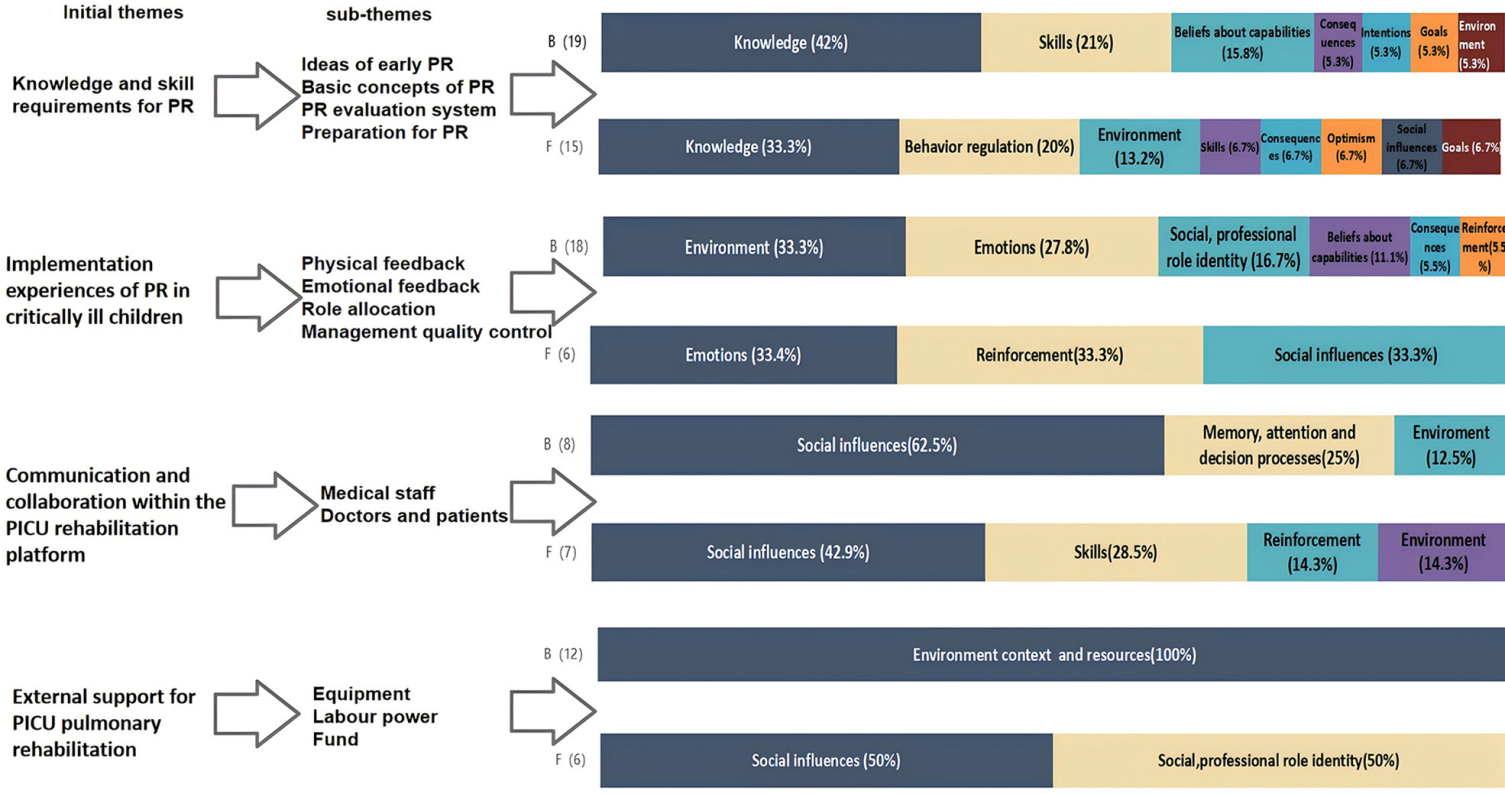
Barriers and Facilitators related to the Theoretical Domain Framework (TDF). B, Barriers; F, Facilitators.

**Figure 2:**
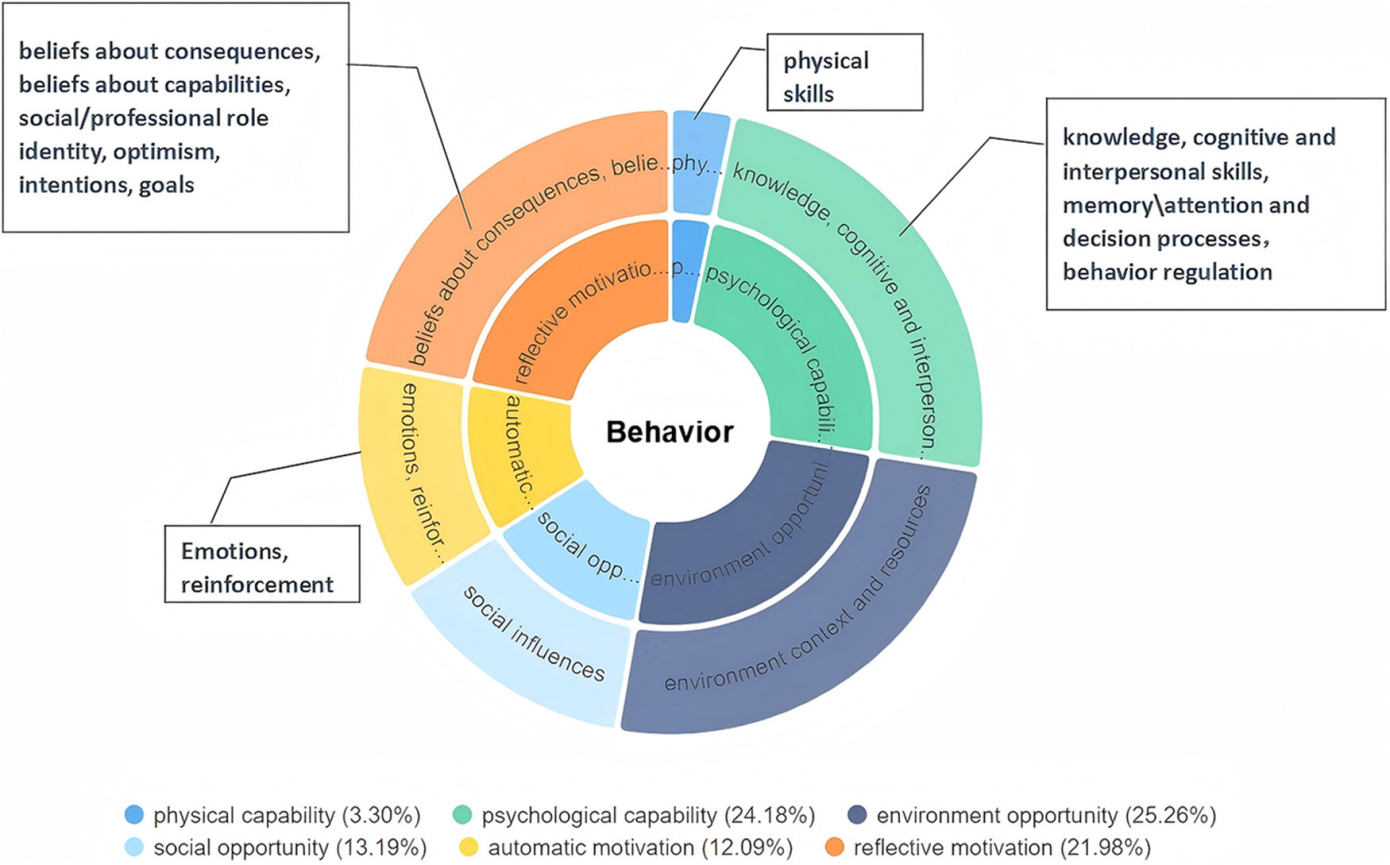
Domains of the TDF within the COM-B model.

The interview results revealed four initial themes and thirteen sub-themes: knowledge and skill requirements for PR, implementation experiences of PR in critically ill children, communication and collaboration within the PICU rehabilitation platform, and external support for PICU pulmonary rehabilitation (see Table 2). These themes encompass all behavioral domains of the TDF and the three main components of the COM-B model (see Figures 1, 2).

**Table 2:** Summary of barriers and facilitators.

Initial themes	Sub themes	Examples of barriers	Examples of facilitators
Knowledge and skill requirements for PR	Ideas of early PR	*N5: The time the child spent on the ventilator was short, and he recovered quickly.* *N5: Doctors don't pay much attention to recovery, either.* *D1: Preserving life is important; recovery is a matter for later once you've survived.* *D2: In the early stages, he couldn't cooperate with the sedatives, but later on, the dosage was reduced, and he could move around on his own in bed. He's not far from being taken off the ventilator, so no additional rehabilitation is needed.* *D4: I've looked into this research, and there are still more studies on adults than on children. The results are inconsistent; some say it benefits lung function, while others say there's no difference. Although I think it should be useful, there's no evidence.*	*N1: Rehabilitation this thing, slow effect, but it is certainly useful, do is certainly good.* *D3: I think pulmonary rehabilitation is beneficial for weaning off the ventilator.* *D5: Why do we advocate early rehabilitation now? Still, there are more and more ventilator-related syndromes, such as ICU-AW and VAP. The purpose of rehabilitation is to reduce their occurrence and improve the prognosis.* *T1: Early rehabilitation is a hot spot now, now there are more and more children in the rehabilitation clinic. We are also trying to promote the ICU, and our director is also very supportive.*
	Basic concepts of PR	*N2: Position change, pat back suction sputum what I still do, other rehabilitation is the doctor and rehabilitation division to do, complex I also won't ah.* *N4: Some doctors do not know that there are some measures such as vibration sputum discharge meter.* *N4: PR contains too many things, usually we do some nursing is pulmonary rehabilitation content, such as some sputum vest, and then our back, such as a variety of positions, but many people do not know that this belongs to pulmonary rehabilitation.* *N6: Has our department talked about the PPT of PR, seems to say, oh, forget.*	*N1: I've seen adult lung rehabilitation, they use breathing instruments with threshold pressure, give him a pressure when inhaling, can exercise the inspiratory strength, we can adjust the parameters of the ventilator to do this.* *N4: As for the recovery of breathing or breathing muscles, I heard that Shanghai is doing it. I think one day our leaders can send us to have a look.* *T3: I often go to read the literature on PR, and if there is relevant training, if I can go, I will apply to our director.*
	PR evaluation system	*N4: I remember in Shanghai, they used the diaphragm electric potential to measure the diaphragm function, but we did not for now.* *N5: It was all decided by the rehabilitation therapist.* *N6: There are individual differences in the doctor's evaluation of children, some of whom do not know whether they need PR, such as prone position, can it be implemented?* *D1: As for the assessment, it's usually done by the rehabilitation department coming over for a consultation and then proceeding with it. I have a rough idea of what the assessment is about, but I'm not very clear on the specifics of how it's done.*	*N5: We have to assess the strength of his respiratory muscles.* *D4: For example, if there is an assessment form for children, who evaluates it, right? There is a specific value, to tell us whether the patient needs to do pulmonary rehabilitation at this time.*
	Preparation for PR	*N1: Pulmonary rehabilitation, we are not as standardized as adults. There is no guidance for children to do this, like guidelines, consensus or something.* *N6: We also went out for further studies, and after a few days of learning, we were given therapist certificates. As for the professionalism, we don't even know if we can go into clinical practice.* *D3: Right now, everything is based on adults, and then we rely on our own clinical experience to figure things out. There's nothing specifically for children. What should we do if we make a mistake?* *D4: What we learned was too superficial and vague, so when it came time to do it, we didn't know where to start.*	*N4: VAP, because now there are SOPs in place, they are all bundled managements, we definitely have to check every day whether these measures are being implemented one by one.* *N5: If you want to implement it, you must have to pass some professional training, or where to go for further study.* *T2: There can be a guiding process, such as what training needs to be done, who is responsible for it, and how long it should take.* *T3: I'm doing clinical research on this recently and want to do a project.*
Implementation experiences of PR in critically ill children	Physical feedback	*N1: For example, he has venous catheterization in his lower limbs, which may affect his functional exercise.* *N3: ICU has acute factors involved; sometimes, his condition may not be able to handle your PR.* *D2: When he goes on the ventilator, will give him sedatives, which he can't cooperate with.* *D5: There are many pulmonary rehabilitation programs that children cannot complete; they don't know how and can't do them.* *T1: For example, if he has a lumbar puncture today, he won't be able to recover for the rest of the day.* *T2:Doing it for babies with a history of epilepsy, they might have seizures, so how can we continue?* *T3: The baby is not willing to do it, they feel tired, no strength.*	*T1: I remember, the last time there was a big girl, she was not the first time in hospital, we gave her a bed bicycle, very cooperative, she knew what to do, she put the foot in.*
	Emotional feedback	*N5: Because nurses sometimes have concerns, if this patient has a risk of fracture and something goes wrong, the nurse will be responsible.* *D2: The older child, he has a psychological condition. For example, if I want to train his respiratory function, I disconnect the ventilator and let him breathe on his own. He becomes very anxious, and then his respiratory function becomes chaotic, and he even stops breathing altogether, holding his breath.* *D5: The ICU environment can be strange for children, especially when they are without their parents. Some children may be restless and uncooperative, while others may appear to comply verbally but do not follow through with actions.* *T1: Throughout the entire process, you must remain highly vigilant, monitor his vital signs, his tubes, and watch for any adverse reactions. Every time I finish a rehabilitation session, I'm drenched in sweat.* *T3: If the experience is not good once, it will be difficult for the baby to cooperate with you next time.*	*N3: Previously, a baby got out of bed with a ventilator, and everyone was overjoyed, rushing over to say “keep it up.”* *T1: The child feels great after riding the bike. Originally, it was just once a day, but later I started doing it for her in the morning and afternoon.*
	Role allocation	*N5: Regarding training for respiratory muscles, such as diaphragm electrical stimulation, this is done by the rehabilitation department. We can't just do it casually; we need to have the proper qualifications, right?* *N6: The boundaries between medical care and rehabilitation are quite blurry. Doctors think I'm only responsible for issuing medical orders, while nurses feel it's extra work. If they do too much, there's no extra pay. The allocation of costs in this area is a big problem that the leadership needs to solve; there's nothing I can do about it.* *D4: We are responsible for issuing the rehabilitation consultation orders, and the implementation is mainly done by nurses and rehabilitation therapists.*	*T2: There can be a guiding process, such as what training needs to be done, who is responsible for it, and how long it should take.*
	Management quality control	*D3: I saw the physiotherapist come over and do it, but they finished it quickly. Is this quality even useful?*	*N6: Like VAP prevention, someone needs to be in charge, and sensitive indicators need to be measured to take responsibility.*
Communication and collaboration within the PICU rehabilitation platform	Medical staff	*N4: Some doctors, when you ask them if this patient needs a rehabilitation consultation, they agree at the moment, but then they turn around and quickly forget.* *N5: The interdisciplinary collaboration is insufficient.* *D3: Every time we apply for something from above, it takes too long. Doesn't this affect the progress of our rehabilitation work?* *T1: We will buy equipment to put in their monitoring room, but the people in PICU don't even know about this equipment.* *T2: We definitely need to receive a consultation request to know which baby needs rehabilitation. If their ICU doesn't issue a medical order, we won't be able to come over and do it.* *T3: Some rehabilitation prescriptions can't be issued, and we have to apply to the higher authorities.*	*N3: In terms of nursing, we might report to the doctor based on the amount of sputum, blood gas analysis results, and ventilator parameters, to discuss whether our child's lungs have more sputum and whether some measures need to be taken.* *N5: We invited the rehabilitation department over to work with them. Originally, for a patient like this, we would have to handle it ourselves. Without professional guidance, it might take us two or three weeks, but now this child was discharged in two weeks.* *N5: Sometimes the director also wants to get the patients moving, but just talking about it isn't enough; we need to establish platforms and create a multidisciplinary mechanism.* *D4: We have a disconnect with the rehabilitation department. Our two departments should hold regular meetings to discuss activity plans and rehabilitation outcomes; otherwise, we are just working separately.* *D5: We often invite some rehabilitation specialists to provide therapy for the children.*
	Doctors and patients	*N2: Many of us are very young children; you can't expect them to engage in physical activity on their own. They not only don't understand but are also very likely unwilling to cooperate.* *T3: Today the child is willing to cooperate with you, but tomorrow he might suddenly refuse to cooperate.*	*D2: Usually, some psychological counseling will be done.* *T3: Some children, if you tell them, “If you pedal the bike more and breathe on your own, you can get this tube out of your mouth sooner and see your mom and dad earlier,” they might be motivated.*
External support for PICU pulmonary rehabilitation	Equipment	*N1: We don't seem to have such a device for respiratory training.* *N4: I remember in Shanghai, they used diaphragm electromyography to measure diaphragm function, but we haven't implemented it here yet.* *N5: The rehabilitation department conducts passive training in bed, focusing on the limbs, but not on the diaphragm.* *N5: After the patient is put on a ventilator, there is a loss of muscle strength, but there is no specific instrument to measure it.* *D2: There is no quantitative assessment. There is a ventilator called NAVA that measures diaphragm potential, but we are currently just applying for it and it is not in use.*	*T1: I am currently researching lung rehabilitation in the ICU. Our director is quite supportive of me; he approves all the equipment applications I submit, and then I bring them to the ICU.* *T3: You can ask the manufacturers for sponsorship; they also need clinical data to support the promotion of their equipment.*
	Labour power	*N2: Our department currently does not have a respiratory therapist.* *N4: Now, because there weren't enough staff and it was quite busy before, and you're managing more patients, under the limited time, it's definitely not going to be executed well.* *D1: It will increase the workload of clinical staff, and if he has too many patients to manage, he won't have time to do it.* *D5: For a lightweight baby, you need at least three people to get him out of bed for activities. We doctors have to supervise, the nurses and therapists help him out of bed, and you say for a heavier one, more people are needed. What about the other patients?*	*N2: I think there should be a professional respiratory therapist.* *N4: First of all, without professionals in this field, he wouldn't know how to proceed, right? Professional matters should be handled by professionals; this way, things should be able to get started.* *N5: Our doctors are not specialized rehabilitation doctors. The ICU actually needs professional respiratory therapists and specialized rehabilitation doctors. Then, they would be completely immersed in the ICU all day, working on this together with the nurses.*
	Fund	*N4: The department has no money; we have to spend money to buy assessment instruments and rehabilitation equipment, right?* *D3: Originally, ICU is quite expensive, so many families are unwilling to proceed with rehabilitation.* *D2: Now the equipment is too expensive; that bed bike was even a gift.*	*T2: Now many rehabilitation programs are covered by health insurance, and the government is very supportive.*

#### 3.2.1 Knowledge and skill requirements for PR

“Knowledge” refers to the concept of early PR for critically ill children and the essential information that medical staff in PICU should possess. It is a multifaceted factor in decision-making behavior and holds a paramount position (42% of the barriers and 33.3% of the facilitators). Interviewees indicated that the primary reason PR is not currently implemented in clinical practice is the absence of relevant theories to guide practical work. There is a significant demand for knowledge regarding the concepts, assessment, and specific practices of early PR. Some interviewees also noted that although they possess a basic understanding of PR, there is minimal training available in PR skills and no certification for qualifications, which hinders the translation of knowledge into practical abilities. The deficiency of evidence-based knowledge and skills has resulted in a lack of confidence in transforming practice. In addition, the proactive efforts of the PICU medical staff to acquire knowledge and skills in PR can facilitate early decision-making behaviors regarding PR recommendations for critically ill children within the department.

#### 3.2.2 Implementation experiences of PR in critically ill children

The experiences of PICU medical staff during the implementation of PR can significantly influence their decision-making behavior regarding future recommendations. Interviewees noted that the objective physical conditions of critically ill children—such as unstable vital signs, the presence of tubes, and the effects of medication—along with subjective emotional states like separation anxiety from parents, fear of the PICU, and concerns about safety risks, all contribute to the challenges of implementing PR. To prevent adverse events, the medical staff decided to postpone the rehabilitation intervention, disregarding the principle of “early.”. At the same time, the ambiguous delineation of responsibilities among doctors, nurses, and therapists impedes the progress of rehabilitation efforts. As assessors and decision-makers, doctors are seldom engaged in the subsequent implementation and evaluation processes. It is worth contemplating whether they can dynamically assess the overall changes in children during the early stages of pulmonary rehabilitation. The nurse who has the most contact with the child is also responsible for a portion of the PR program. An additional consideration is whether the nurse should assume the responsibilities of the rehabilitation therapist in their absence.

#### 3.2.3 Communication and collaboration within the PICU rehabilitation platform

“Social influence” includes social support and group conflict, which are mixed factors that affect recommendation decision-making behavior and hold an important position (62.5% of barriers and 42.9% of facilitators). Interviewees indicated that the multidisciplinary collaboration platform for PR is inadequate. They also noted that information sharing is insufficient and that there are discrepancies in understanding among various specialties. These factors make it challenging to develop the most effective pulmonary rehabilitation plan for the children. Additionally, due to the young age of children, there are differences in cognition and communication compared to adults, which also impact the implementation of PR.

#### 3.2.4 External support for PICU pulmonary rehabilitation

The limitations of hardware, professionals, and funding are significant factors that impact early PR in critically ill children. The majority of interviewees felt that the PICU has a high workload and that basic nurse duties, drug administration, and invasive treatments take precedence over rehabilitative exercises. There is a high expectation for having a professional rehabilitation team stationed in PICU to provide more specialized pulmonary rehabilitation activities for the children.The interviewees also said that the number of existing rehabilitation equipment was small to support the implementation of some rehabilitation activities, and the operation was complex. They hoped that there would be more labor-saving and convenient methods to help children complete the training.

## 4 Disscussion

### 4.1 Changing perceptions, increasing PICU medical staff's early PR awareness and evidence-based skills

Traditionally, the focus of treatment in the PICU has been on resuscitation, the management of critical illness processes, and the reversal of organ failure.Therefore, critically ill children often use sedatives and are bedridden for long periods due to the need for safety, comfort, and hemodynamic stability.In recent years, early pulmonary rehabilitation during intensive care has become increasingly common. PICU medical staff are participants and guides in pulmonary rehabilitation, and their knowledge and professional skill levels directly influence the implementation of pulmonary rehabilitation in PICU.According to the study's findings, medical staff, nurses, and technicians all have some knowledge about PR, but their lack of comprehensive knowledge and high-level, evidence-based medical support has made them less inclined to advocate for early PR for children in critical condition. This is consistent with the findings of Nardo et al.'s quantitative study (15). PICU medical staff also lack systematic training and evaluation, which results in a lack of competence and trust in their ability to perform. All medical personnel involved in patient care should get formal pulmonary rehabilitation training, according to the American Thoracic Society's policy statement on enhancing the implementation, use, and supply of pulmonary rehabilitation (16). Therefore, on the one hand, it is necessary to strengthen research and evidence-based training to increase PICU medical staff's awareness of evidence-based practice. This can be achieved through high quality clinical research, continuous practice and validation to increase the level of evidence, and the development of clinical practice guidelines. On the other hand, various forms of knowledge and skills training in early PR for critically ill children should be implemented to deepen the understanding and application of early PR and to continuously improve cognitive levels and evidence-based practice skills.

### 4.2 Stimulate motivation, enhance the willingness of PICU medical staff to recommend early PR

PICU medical staff's willingness to recommend PR is diminished by a lack of self-efficacy stemming from insufficient skills and a lack of intrinsic motivation influenced by external environmental factors. Therefore, effective intervention strategies should be implemented to enhance behavioral motivation (1). Changes in motivation are a process of calculating risks, and risk-benefit analysis can influence motivation (17). Once individuals acquire the ability to develop, they should create a targeted action plan grounded in a comprehensive understanding of the risks and rewards associated with their proposed decisions. They should implement a monitoring system, enhance the effectiveness of rewards and punishments, and strengthen positive experiences. This approach will empower individuals to transition from thinking to execution of the action plan, consistently derive positive outcomes, and confidently engage in their practice (2). Research indicates that PICU medical staff derive a sense of professional value and recognition from patients' recovery and affirmation from their peers. This recognition, in turn, positively influences their professional behavior and fosters the development of their skills (18). Therefore, managers should proactively identify best practices for early pulmonary rehabilitation in PICU and effectively guide and motivate the remaining staff. This approach will enhance the willingness and enthusiasm of the PICU team to implement early pulmonary rehabilitation (3). Clarify the roles and responsibilities of each discipline, establish workflows and protocols, and ensure the smooth progress of pulmonary rehabilitation work.

### 4.3 Optimize the environment, improve the atmosphere for early PR recommendations in PICU

The PICU environment can influence the prevalence of lung rehabilitation. Firstly, in previous studies, nursing staff play a central role in rehabilitation activities, with nurses conducting 37% to 48% of rehabilitation activities (19, 20). However, in this study, nurses may delay or refuse to implement lung rehabilitation due to staff shortages and heavy workloads. In addition, there is a negative correlation between nurses' work-related stress and patient safety; as stress levels increase, the likelihood of adverse events and disputes also rises (21). Therefore, determining how to adjust the existing staffing structure of the PICU in the future to achieve optimal human resource allocation is a pressing challenge that managers must address. Secondly, fostering a culture of multidisciplinary collaboration in the PICU is essential for the project's success (22). The implementation of early PR depends not only on nurses but also necessitates the active participation of physicians, rehabilitation therapists, respiratory therapists, and other disciplines. Research indicates that utilizing a Multidisciplinary Team (MDT) can enhance the implementation rate of rehabilitation programs and improve rehabilitation outcomes (23). This study emphasizes the urgent need for multidisciplinary collaboration platforms. Furthermore, the current lack of equipment hampers existing PR activities, and the training methods available are insufficient. Therefore, managers should proactively introduce advanced rehabilitation equipment to enhance the efficiency of PR activities. Finally, it is essential to further improve the medical system and support related to children's rehabilitation in the future, instilling greater confidence in medical staff and the families of patients, and encouraging their commitment to recommending early pulmonary rehabilitation.

## 5 Conclusion

This study examines the barriers and facilitators influencing decision-making behavior related to early lung rehabilitation recommendations among medical staff in PICU. Utilizing the COM-B theory and the TDF, data were collected across four main themes and thirteen sub-themes. The identified barriers and facilitators were categorized into fourteen TDF domains and three COM-B modules. This qualitative analysis addresses existing research gaps and can serve as a valuable reference for the development and implementation of early pulmonary rehabilitation intervention strategies for pediatric patients in the PICU in the future. The current study excluded children with severe medical conditions; subsequent research should consider conducting interviews with this population to obtain valuable insights into their experiences with pulmonary rehabilitation. This approach would enhance the understanding necessary to confidently advocate for lung rehabilitation in the future.

## Data Availability

The original contributions presented in the study are included in the article/Supplementary Material, further inquiries can be directed to the corresponding authors.
