# Supplementary material for: Early pulmonary rehabilitation recommended decision-making behavior experience among pediatric intensive care unit medical staff: a qualitative study
Source: Front Pediatr. 2025 May 22;13:1535459. doi: 10.3389/fped.2025.1535459 (PMC12137239; doi:10.3389/fped.2025.1535459)
Supplement: Supplementary file 1 [file Datasheet1.docx]

Supplementary Material

- **Standards for Reporting Qualitative Research (SRQR)***

<http://www.equator-network.org/reporting-guidelines/srqr/>

**Page/line no(s).**

**Title and abstract**

| **Title** - Concise description of the nature and topic of the study Identifying the  study as qualitative or indicating the approach (e.g., ethnography, grounded  theory) or data collection methods (e.g., interview, focus group) is recommended | Page 1/ Title |
| --- | --- |
| **Abstract** - Summary of key elements of the study using the abstract format of the intended publication; typically includes background, purpose, methods, results,  and conclusions | Page 2 |

**Introduction**

| **Problem formulation** - Description and significance of the problem/phenomenon studied; review of relevant theory and empirical work; problem statement | Page 2, 3/ Introduction |
| --- | --- |
| **Purpose or research questio**n - Purpose of the study and specific objectives or questions | Page 3/ Introduction line 78-80 |

**Methods**

| **Qualitative approach and research paradigm** - Qualitative approach (e.g.,  ethnography, grounded theory, case study, phenomenology, narrative research) and guiding theory if appropriate; identifying the research paradigm (e.g.,  postpositivist, constructivist/ interpretivist) is also recommended; rationale** | Page 3/ 2.1, 2.2 |
| --- | --- |
| **Researcher characteristics and reflexivity** - Researchers’ characteristics that may influence the research, including personal attributes, qualifications/experience, relationship with participants, assumptions, and/or presuppositions; potential or actual interaction between researchers’ characteristics and the research  questions, approach, methods, results, and/or transferability | Page 4/ 2.4, line 121-125 |
| **Context** - Setting/site and salient contextual factors; rationale** | Page 4/ 2.3 |
| **Sampling strategy** - How and why research participants, documents, or events  were selected; criteria for deciding when no further sampling was necessary (e.g., sampling saturation); rationale** | Page 4/ 2.3 |
| **Ethical issues pertaining to human subjects** - Documentation of approval by an appropriate ethics review board and participant consent, or explanation for lack thereof; other confidentiality and data security issues | Page 3/ 2.1, line86, 87 |
| **Data collection methods** - Types of data collected; details of data collection  procedures including (as appropriate) start and stop dates of data collection and analysis, iterative process, triangulation of sources/methods, and modification of procedures in response to evolving study findings; rationale** | Page 4/ 2.4, line 125-130 |
| **Data collection instruments and technologies** - Description of instruments (e.g., interview guides, questionnaires) and devices (e.g., audio recorders) used for data collection; if/how the instrument(s) changed over the course of the study | Page 4/ 2.4, line 131-139/ [Supplementary Material](https://www.frontiersin.org/guidelines/author-guidelines#supplementary-material) |
| **Units of study** - Number and relevant characteristics of participants, documents, or events included in the study; level of participation (could be reported in results) | Page 5/ 3.1,table 1 |
| **Data processing** - Methods for processing data prior to and during analysis,  including transcription, data entry, data management and security, verification of  data integrity, data coding, and anonymization/de-identification of excerpts | Page 5/ 2.5 |
| **Data analysis** - Process by which inferences, themes, etc., were identified and  developed, including the researchers involved in data analysis; usually references a  specific paradigm or approach; rationale** | Page 5/ 2.5 |
| **Techniques to enhance trustworthiness** - Techniques to enhance trustworthiness  and credibility of data analysis (e.g., member checking, audit trail, triangulation); | Page 5/ 2.5, line 150-151 |

**Results/findings**

| **Synthesis and interpretation** - Main findings (e.g., interpretations, inferences, and themes); might include development of a theory or model, or integration with  prior research or theory | Page 6, 7/ 3.2/ figure 1,figure 2 |
| --- | --- |
| **AaLinks to empirical data** - Evidence (e.g., quotes, field notes, text excerpts, photographs) to substantiate analytic findings |  |

**Discussion**

| **Integration with prior work, implications, transferability, and contribution(s) to the field -** Short summary of main findings; explanation of how findings and  conclusions connect to, support, elaborate on, or challenge conclusions of earlier scholarship; discussion of scope of application/generalizability; identification of unique contribution(s) to scholarship in a discipline or field | Page 14-15/ 4. dicussion |
| --- | --- |
| **Limitations** - Trustworthiness and limitations of findings |  |

**Other**

| **Conflicts of interest** - Potential sources of influence or perceived influence on study conduct and conclusions; how these were managed | Page 16/ line 301 |
| --- | --- |
| **Funding** - Sources of funding and other support; role offunders in data collection, interpretation, and reporting | Page 16/ line 310 |

| *The authors created the SRQR by searching the literature to identify guidelines, reporting standards, and critical appraisal criteria for qualitative research; reviewing the reference lists of retrieved sources; and contacting experts to gain feedback. The SRQR aims to improve the transparency of all aspects of qualitative research by providing clear standards for reporting qualitative research.  **The rationale should briefly discuss the justification for choosing that theory, approach, method, or technique rather than other options available, the assumptions and limitations implicit in those choices, and how those choices influence study conclusions and transferability. As appropriate, the rationale for several items might be discussed together. |
| --- |

**Reference:**

O'Brien BC, Harris IB, Beckman TJ, Reed DA, Cook DA. **Standards for reporting qualitative research: a synthesis of recommendations.** *Academic Medicine*, Vol. 89, No. 9 / Sept 2014 DOI: 10.1097/ACM.000000000000038

- **Semi-Structured Interview Guide**

1. Can you talk about your understanding of pulmonary rehabilitation? How did you learn about pulmonary rehabilitation?
2. Do you think children in the PICU can undergo early pulmonary rehabilitation? Is it necessary? Please discuss your reasons for this belief.
3. In your daily work in the PICU, have you implemented early pulmonary rehabilitation for patients? How specifically was it implemented? What were your feelings during the implementation process?
4. Did you encounter any difficulties in deciding to implement or during the implementation of pulmonary rehabilitation? How did you resolve them?
5. What factors do you think hinder early pulmonary rehabilitation for children in the PICU?
6. What factors do you think promote early pulmonary rehabilitation for children in the PICU?
7. In the future, what support and improvements would you like to receive to better carry out early pulmonary rehabilitation for children in the PICU?
8. Is there anything else you would like to share about early pulmonary rehabilitation for children in the PICU?

**
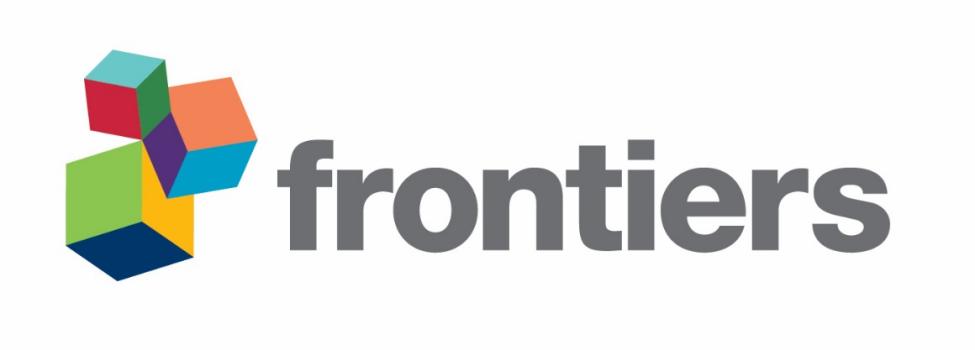
**
